# Femoral hernia with an incarcerated appendix epiploica mimicking a hydrocele of the canal of Nuck: a case series

**DOI:** 10.1093/jscr/rjag206

**Published:** 2026-03-29

**Authors:** Akari Sonoda, Akihiko Togashi, Tetsuya Okino, Chitoshi Ohara

**Affiliations:** Department of Gastroenterological Surgery, NHO Kumamoto Saishun Medical Center, Koshi, Kumamoto 861-1196, Japan; Department of Gastroenterological Surgery, NHO Kumamoto Saishun Medical Center, Koshi, Kumamoto 861-1196, Japan; Department of Gastroenterological Surgery, NHO Kumamoto Saishun Medical Center, Koshi, Kumamoto 861-1196, Japan; Department of Gastroenterological Surgery, NHO Kumamoto Saishun Medical Center, Koshi, Kumamoto 861-1196, Japan

**Keywords:** femoral hernia, appendix epiploica, hydrocele of the canal of Nuck, laparoscopic surgery

## Abstract

In women, inguinal hernias that involve incarceration of intraperitoneal tissues can cause a hydrocele, which can be difficult to distinguish from a hydrocele of the canal of Nuck (HCN). We describe two cases in which an appendix epiploica (AE) became incarcerated in a femoral hernia, causing a hydrocele. In both cases, computed tomography indicated HCN as a differential diagnosis. Ultrasonography revealed protruding adipose tissue within the inguinal hydrocele, which was considered different from HCN. We performed laparoscopic surgery to diagnose, and found the AE incarcerated in femoral hernia. We released the AE, then the inguinal hydrocele completely disappeared. We performed transabdominal preperitoneal repair using mesh, and both patients experienced a favorable postoperative course. Inguinal hydrocele may be due to incarceration of an AE into a femoral hernia. By combining computed tomography and ultrasonography, the appropriate diagnosis and surgical procedure can be selected.

## Introduction

Incarceration of an appendix epiploica (AE) into a femoral hernia sac is very rare. Inguinal hernias are common, but femoral hernias account for only approximately 4%–7% of inguinal hernias [1, 2]. Compared with men, fewer women are diagnosed with inguinal hernias, but femoral hernias are more common in middle-aged and older women, and the rate of incarceration is high at 30%–50% [3, 4]. While the small intestine, large intestine, and greater omentum commonly become incarcerated in hernias, there have also been reports of incarceration of the appendix and fallopian tubes [5]. When something other than the intestine becomes incarcerated and causes edema in the inguinal region in women, it can be difficult to differentiate the hernia from a hydrocele of the canal of Nuck (HCN). Herein, we report two cases in which we used computed tomography (CT) and ultrasonography to differentiate HCN from an incarcerated AE in a femoral hernia and performed transabdominal preperitoneal repair (TAPP).

## Case series

### Case 1

An 88-year-old woman visited our hospital with a 4-cm swelling in her right groin that had been increasing in size for several days. On presentation, she had no pain or redness at the swelling site, and laboratory test results were within normal limits.

**Figure 1:**
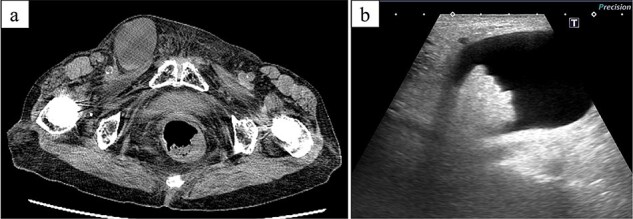
CT and ultrasonographic findings. (a) CT image showing a 4-cm right inguinal hydrocele. (b) Ultrasonography showing protruding fatty tissue within the hydrocele.

CT revealed a cystic lesion in her right groin, which was diagnosed as an HCN or an inguinal hernia (Fig. 1a). Ultrasonography revealed adipose tissue protruding from the deep (peritoneal) portion of the hydrocele (Fig. 1b). Laparoscopic surgery was performed 8 days after the patient visited our hospital.

**Figure 2:**
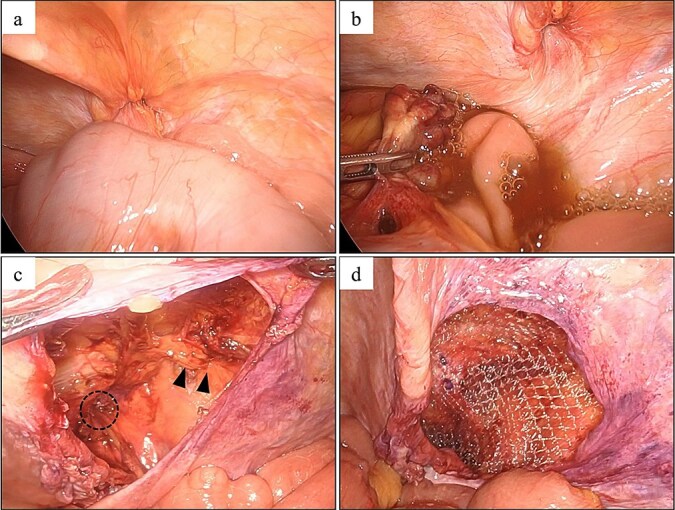
Laparoscopic findings in Case 1. (a) AE of the transverse colon incarcerated in a right femoral hernia. (b) The AE was retracted from the hernia, after which clear ascites was released. (c) The round ligament of the uterus (arrowhead) and the femoral hernia orifice (circle) were identified. (d) Intraoperative image showing hernia repair using a 3DMax mesh (CR bard, Inc., Murray Hill, NJ, USA).

We diagnosed an incarcerated AE of the transverse colon into a femoral hernia (Fig. 2). We gently extracted the incarcerated AE, after which clear yellow ascites flowed into the abdominal cavity, and the inguinal swelling completely disappeared. Although the top of the extracted AE was partially dark red, suggesting incomplete blood flow disorder, we determined that there was no infection and decided to perform mesh repair. We performed TAPP, and the femoral hernia was repaired using a 3DMax mesh (size M: 8.5 × 13.7 cm; CR Bard, Inc., Murray Hill, NJ, USA). The patient experienced a good postoperative course.

### Case 2

An 80-year-old woman was referred to our institution with a suspected incarcerated hernia that presented as a 4-cm mass in the left groin. She had no pain or redness at the affected site, and laboratory test results were within normal limits. Manual mass reduction was difficult. Emergency ultrasonography revealed a cystic lesion in the groin and deep fatty tissue, similar to the findings in Case 1.

**Figure 3:**
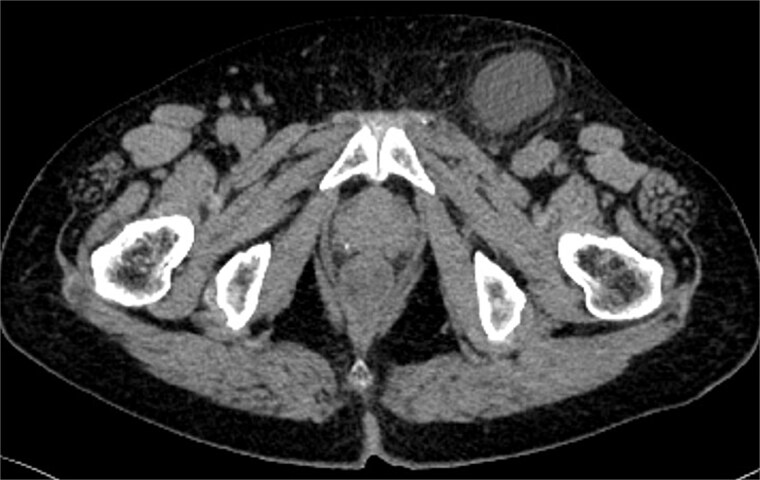
CT findings in Case 2. CT image showing a 4-cm left inguinal hydrocele.

A radiologist interpreted the CT findings and made a diagnosis of HCN (Fig. 3). On the basis of our experience with Case 1, we determined that emergency surgery was unnecessary and performed laparoscopic surgery 15 days after the patient’s presentation.

**Figure 4:**
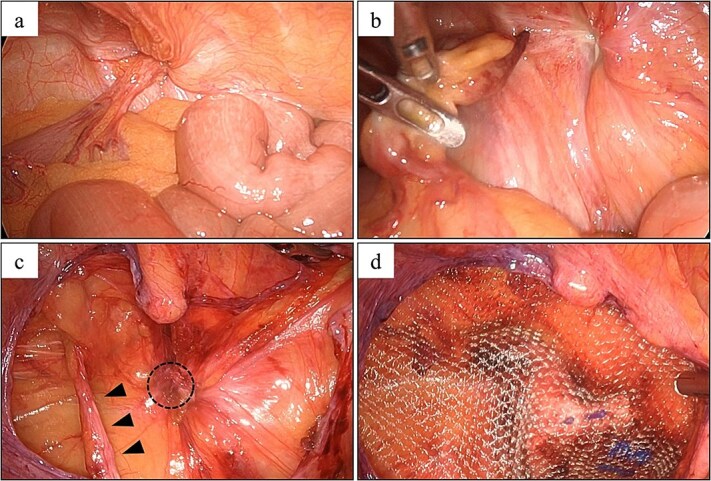
Laparoscopic findings in Case 2. (a) An AE of the sigmoid colon was incarcerated in a left femoral hernia. (b) The AE was retracted from the hernia, after which clear ascites was released. (c) The round ligament of the uterus (arrowhead) and femoral hernia orifice (circle) were identified. (d) Hernia repair using a 3DMax mesh (CR bard Inc., Murray Hill, NJ, USA).

Intraoperatively, we identified an AE of the sigmoid colon incarcerated in a left femoral hernia (Fig. 4). After release of the incarcerated AE, the inguinal swelling completely disappeared. Although there were signs of compromised blood flow at the top of the AE, the ascites was clear, and we determined that there was no infection. We performed TAPP and repaired the femoral hernia using a 3DMax mesh, size M (8.5 × 13.7 cm; CR Bard, Inc.). The patient experienced a good postoperative course.

## Discussion

Inguinal hernias are common causes of groin swelling. The most common subtypes are indirect inguinal, and direct inguinal and femoral hernias; 90% of inguinal hernias occur in men, and 75% occur in women [6]. Femoral hernias have a high incidence of incarceration due to a small hernia orifice, with most cases involving incarceration of the small intestine, large intestine, or omentum. However, there are reported cases of incarceration involving an AE of the colon, and among these, most cases involved AE of the sigmoid colon penetrating into a left inguinal hernia [7, 8]. To our knowledge, few cases of AE incarceration of a right-sided femoral hernia treated with one-stage TAPP surgery have been reported. In both of our cases, ultrasonography clearly demonstrated protrusion of adipose tissue within the hydrocele, suggesting incarceration. Although incarceration of an AE into a femoral hernia is rare, combining various imaging modalities may be useful for diagnosis.

HCN is a rare condition defined as an inguinal hydrocele that occurs along the round ligament of the uterus [9]. There are two types of HCN: communicating, which communicates with the abdominal cavity and changes in size, and non-communicating, which does not change in size. Because of the developmental process, HCNs often coexist with inguinal hernias [9]. In both of our cases of inguinal hydrocele, there was no change in size, and manual reduction was impossible. Considering the CT findings, HCN was the most likely diagnosis. Generally, treatment for HCN involves complete excision of the hydrocele. For HCNs located close to the body surface, a laparoscopic approach alone is likely to be insufficient [10]. In both cases, because the ultrasonographic findings were not typical of HCN [11, 12], we decided to perform laparoscopy and diagnosed an incarcerated femoral hernia. The hernia orifice was located away from the round ligament of the uterus, and after releasing the incarceration, the hydrocele completely disappeared. HCN was not present in either case.

In incarcerated inguinal hernia surgery, if there are signs of intestinal necrosis or infection, primary mesh repair is inappropriate and secondary repair is recommended [13]. In our cases, although blood flow was impaired in part of the AE, infection was not detected. Therefore, primary mesh repair was performed, and the patients progressed smoothly postoperatively, without infectious complications.

**Figure 5:**
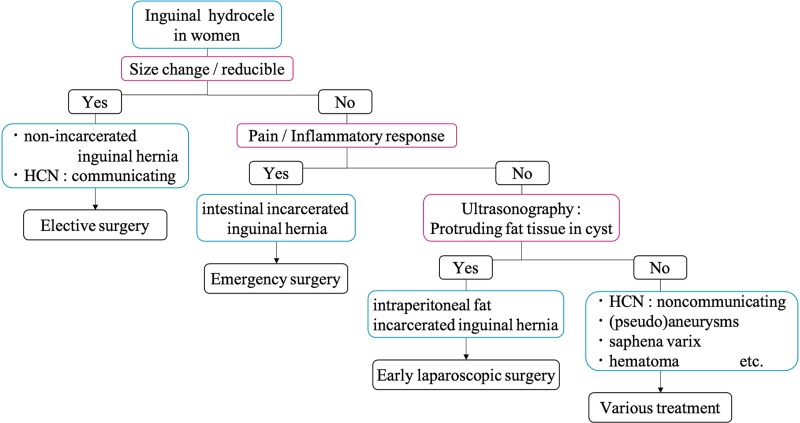
Flowchart of the differential diagnosis for inguinal hydrocele in women.

The appropriate timing of surgery for patients with similar conditions has not been established because of the limited number of cases. In our cases, on the basis of physical examination findings and blood test results, we did not perform emergency surgery. The first patient underwent surgery 8 days after presentation, and the second patient underwent surgery 15 days after presentation; both had uneventful postoperative courses. However, considering the risk of progressive blood flow impairment in the AE leading to necrosis and infection, early surgery is recommended [14]. We have created a flowchart (Fig. 5) to help differentiate of HCN from other diseases in women with inguinal hydrocele, which we believe will be helpful in clinical practice [15].

In conclusion, we reported two rare cases in which an AE of the colon was incarcerated in a femoral hernia, making it difficult to differentiate femoral hernia from HCN. Ultrasonography was useful for the differential diagnosis, and laparoscopic surgery yielded favorable treatment outcomes.

## Data Availability

The data reported in this article are unavailable to other researchers because of the need to maintain patient privacy.
